# Lipid droplets as multifunctional organelles related to the mechanism of evasion during mycobacterial infection

**DOI:** 10.3389/fcimb.2023.1102643

**Published:** 2023-02-23

**Authors:** Patrícia Elaine de Almeida, Núbia Maria Pereira de Sousa, Pollianne Garbero Rampinelli, Renata Vieira de Sousa Silva, José Raimundo Correa, Heloisa D’Avila

**Affiliations:** ^1^ Laboratory of Cellular Biology, Department of Biology, Federal University of Juiz de Fora (UFJF), Juiz de Fora, Minas Gerais, Brazil; ^2^ Laboratory of Microscopy and Microanalysis, Department of Cell Biology, University of Brasilia, Brasilia, DF, Brazil

**Keywords:** lipid metabolism, lipid droplets, mycobacteria, tuberculosis, phagosome

## Abstract

Tuberculosis (TB) is an infectious disease caused by the bacteria of the *Mycobaterium tuberculosis* (*Mtb*) complex. The modulation of the lipid metabolism has been implicated in the immune response regulation, including the formation of lipid droplets (LD)s, LD-phagosome association and eicosanoid synthesis. *Mtb*, *M. bovis* BCG and other pathogenic mycobacteria, as well as wall components, such as LAM, can induce LDs formation in a mechanism involving surface receptors, for instance TLRs, CD36, CD14, CD11b/CD18 and others. In addition, the activation of the lipid-activated nuclear receptor PPARγ is involved in the mechanisms of LD biogenesis, as well as in the modulation of the synthesis of lipid mediators. In infected cells, LDs are sites of compartmentalized prostaglandin E_2_ synthesis involved in macrophage deactivation, bacterial replication and regulation of the host cytokine profile. LDs also have a function in vesicle traffic during infection. Rab7 and RILP, but not Rab5, are located on LDs of infected macrophages, suggesting that LDs and phagosomes could exchange essential proteins for phagosomal maturation, interfering in mycobacterial survival. The pharmacological inhibition of LDs biogenesis affects the bacterial replication and the synthesis of lipid mediators and cytokines, suggesting that LDs may be new targets for antimicrobial therapies. However, it is still controversial if the accumulation of LDs favors the mycobacterial survival acting as an escape mechanism, or promotes the host resistance to infection. Thus, in this mini-review we discuss recent advances in understanding the important role of LDs in the course of infections and the implications for the pathophysiology of mycobacteriosis.

## Introduction

Pathogenic mycobacteria, including *Mycobacterium tuberculosis* (*Mtb*), *Mycobacterium leprae*, and the attenuated vaccine strain have an efficient set of adaptations that support crucial infection events involved in many approaches to subvert host cellular functions (Colonne et al., 2016; Barisch and Soldati, 2017). Moreover, the fact that approximately a quarter of the world’s population is infected with *Mtb* demonstrates a robust well-adapted long-term interaction between mycobacteria and their host (WHO, 2019). Once the bacilli are inhaled, alveolar macrophages become infected with *Mtb* and differentiate into foamy macrophages leading to lung inflammation.

**Figure 1:**
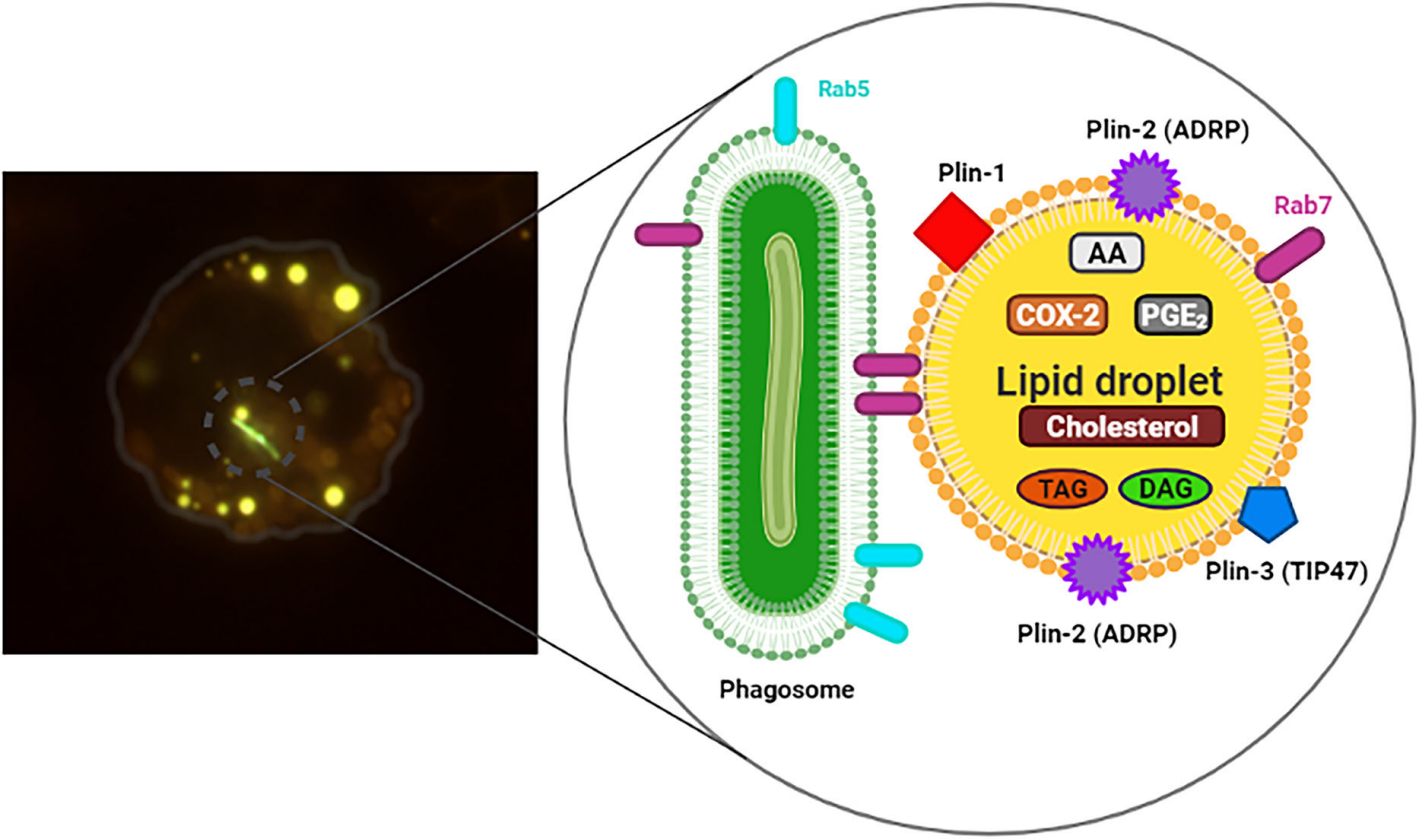
Infected macrophage exhibiting Lipid droplets (LDs). On the left, murine macrophage showing internalized mycobacteria (*M. bovis* BCG) stained with Live/Dead Baclight Bacterial^®^ Viability Kit (green) and many cytoplasmic LDs stained with Bodipy^®^ 493/503 (yellow). In the detail, representation of the structure and composition of LD in association with phagosome containing bacteria (green), Rab5 and Rab7. Triacylglycerol (TAG), diacylglycerol (DAG), prostaglandin E_2_ (PGE_2_), cyclooxygenase-2 (COX-2), arachidonic acid (AA), structural proteins: Plin-1, Plin-2 (ADRP) and Plin-3 (TIP47).

In mycobacteria infection, the foam aspect of macrophages is a reflection of the intracellular lipid accumulation or of lipid droplets (LD)s biogenesis (**Figure 1**). LDs can form a first-line intracellular defense in macrophages. They act as a molecular switch in innate immunity, responding to pathogens signals by both reprogramming cell metabolism and eliciting protein-mediated antimicrobial mechanisms (Laval et al., 2021; Zhang et al., 2021).

The newly formed LDs during mycobacterial infections is a regulated phenomenon and involves activation of membrane receptors during the host response. Different members of the Toll like receptors (TLR) family, including TLR1, TLR2, TLR4 and TLR6 have been implicated in mycobacterial infection recognition (Takeuchi and Akira, 2002; D’Avila et al., 2006; Mattos et al., 2011a; Almeida et al., 2014). TLR2 signaling has a key role in the activation of the peroxisome proliferator-activated receptors (PPAR)γ and accumulation of LDs macrophages during mycobacteria infection (Almeida et al., 2009).

Downstream pathways involved in LDs biogenesis were shown to induce the activation of transcription factors including regulators of the lipid metabolism and nuclear receptors, such as PPARs, liver X receptor (LXR), SREBPs, and HIF (Szatmari et al., 2007; Mei et al., 2009; Knight et al., 2018). The PPARγ transcription factor directly regulates the expression of several genes participating in fatty acid uptake, lipid storage and inflammatory response by binding to specific DNA response elements in target genes such as heterodimers with the retinoid X receptors (RXR), fatty acid synthase (FAS) and perilipin-2 (PLIN)-2 (Gearing et al., 1993; Fujimoto et al., 2001).

In fact, LDs can represent an important escape mechanism for pathogenic bacteria such as *Mtb*, BCG, *M. leprae*, as well as *Chlamydia trachomatis; Chlamydia muridarum* and *Salmonella typhimurium* in different models (D’Avila et al., 2006; Cocchiaro et al., 2008; Peyron et al., 2008; Fujimoto and Parton, 2011; Mattos et al., 2011a, b; Rank et al., 2011; Souza et al., 2022). During the bacterial infection, LDs were found in close proximity, inside of phagosomes or in the lumen of the pathogen containing vacuole of macrophages. This association depends on specific combinations of both, host cell and pathogen components (Cocchiaro et al., 2008; Daniel et al., 2011; Olzmann and Carvalho, 2019). Although LDs can come into intimate contact with pathogen-containing phagosomes, the mechanism by which this phenomenon occurs remains poorly understood.

The mycobacteria interfere with host trafficking pathways by modulating events in phagosomal maturation through the handling of the specific metabolic pathways, thus creating a protected niche, which facilitates nutrients access and favors the establishment of infection (Barisch and Soldati, 2017; Pereira-Dutra et al., 2019; Roque et al., 2020). Roque et al., demonstrated that the interaction between LDs and mycobacteria-containing phagosomes initially occurs in a mechanism dependent of bacterial cell wall components, such as LAM and PIM. Furthermore, this interaction promotes the accumulation of late endosome protein Rab7 and its effector RILP at LDs, which mediates LD-phagosome interactions in macrophages (Roque et al., 2020). Thus, we suggest that LDs could be a safe shelter because they preserve the bacilli and constitute an important source of nutrients.

However, it needs to be elucidated if the foam macrophage survival versus death by apoptosis, pyroptosis or necrosis which has been observed following macrophage infection with virulent *Mtb* is beneficial to the establishment of the infection (Divangahi et al., 2013). Moreover, the signalling pathways that regulate LDs biogenesis during mycobacterial infection and their contribution to the pathophysiology of TB are not fully established. Thus, this review will focus on the latest understanding of the adaptation and regulation of the different lipid related pathways during infection and on the interactions between the mycobacteria and the host lipid metabolisms during the infection process.

## Differential downstream signaling pathways activation during pathogenic and non-pathogenic infection

The LDs biogenesis is a phenomenon that can be rapidly induced after a short time of stimulation with PAMPS, such as LPS and LAM. It has been demonstrated that this phenomenon depends not only on the direct interaction between the pathogen and the host cells, but also on the indirect mechanisms of bystander amplification induced system through bacterial components and/or host generated inflammatory mediators (D’Avila et al., 2006; Peyron et al., 2008; Almeida et al., 2009).

It has been demonstrated that PPARγ activation by mycobacterial infection has a key role in LDs biogenesis (Almeida et al., 2009; Rajaram et al., 2010). Moreover, the decreased production of proinflammatory cytokines and NO could contribute to a favorable environment for pathogens, thereby suggesting that mycobacterial-induced PPARγ expression and LD biogenesis may act as an escape mechanism for this intracellular parasite (Almeida et al., 2014). Following PPARγ knockdown, macrophages had significantly better ability to control *Mtb* growth, as assessed by colony forming assays (Rajaram et al., 2010). The increased control of mycobacterial infection by PPARγ inhibition was concomitant with an increase in TNFα synthesis and a decreased LDs biogenesis (Almeida et al., 2009; Rajaram et al., 2010), providing evidence that mycobacterial-induced PPARγ is an important mechanism in favoring mycobacterial growth in macrophages, at least partly through transcriptional regulation of inflammatory cytokines and lipid metabolism. Collectively, these findings suggest that mycobacteria utilize PPARγ signaling as an escape mechanism that enables survival within the hostile environment of macrophages.

**Figure 2:**
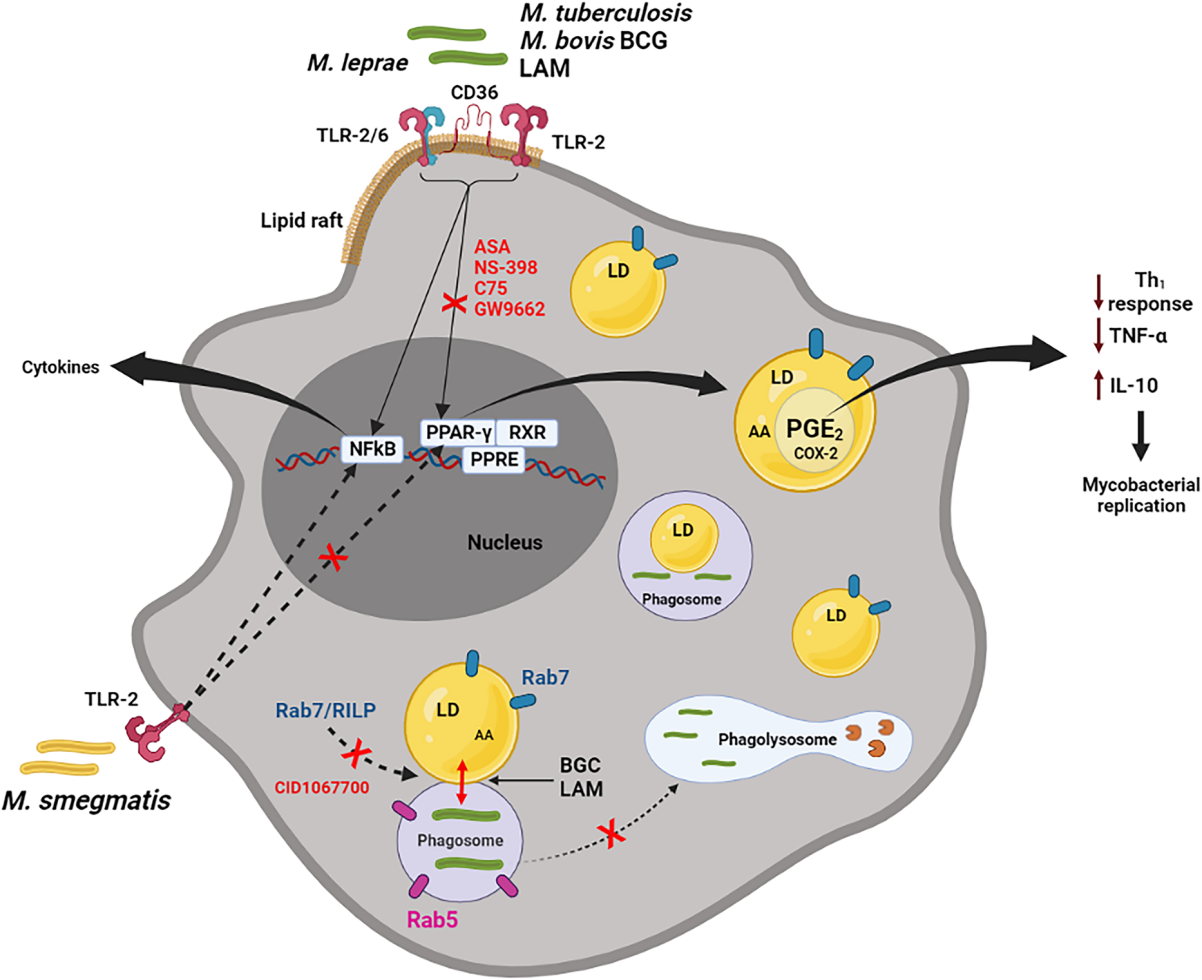
Macrophage activation by pathogenic mycobacteria. *M. bovis*, *Mtb*, *M. leprae* and LAM (but not *M. smegmatis*) can active macrophages by membrane receptors, such as TLR-2, TLR2/6, CD36 localized in lipid rafts, inducing LDs formation, through PPARγ activation. This pathway can be inhibited by ASA (aspirin), NS-398, C75 and GW9662 (selective PPARγ antagonist). Through NF-κB signaling, pathogenic and non-pathogenic mycobacteria induce the synthesis of cytokines, such as TNFα. LDs, presenting substrate (AA) and enzyme (COX-2), are sites for compartmentalized PGE_2_ synthesis. PGE_2_ is a potent eicosanoid which reduces the host Th1 immune response (through inhibition of the TNFα and induction of the IL-10), collaborating with the replication of the bacteria. LAM and BCG actively induce phagosome and LDs interaction in a Rab7 and RILP-dependent mechanism. This interaction can be reduced by the pan GTPase Rab7 inhibitor, CID1067700. Rab7, but not Rab5, is frequently observed on LDs induced by BCG. In general, LDs may be involved in the escape mechanism during mycobacterial infection, through modulation of inflammatory mediator synthesis and the endocytic pathway (could be preventing the maturation phagosome to phagolysosome). Hydrolytic enzymes in the phagolysosome are represented by pacman image (in brown).

In this scenario, TLR2 activation in macrophages appears to be critical for mycobacteria recognition as well as being classically recognized as a principal inducer of signals in mycobacterial infection (Heldwein and Fenton, 2002; Jo et al., 2007). BCG and the purified cell wall component from *Mtb*, such as LAM and PIM, are potent inducers of LDs biogenesis in leukocytes. However, BCG and LAM from *Mtb* failed to induce LDs biogenesis in TLR2, but not TLR4 or TLR6, knockout (KO) mice, suggesting an important role for TLR2 in this phenomenon (**Figure 2**) (D’Avila et al., 2006; Almeida et al., 2009; Roque et al., 2020).

Interestingly, activation of macrophages *in vitro* with a non-pathogenic *M. smegmatis* (**Figure 2**), zymosan or Pam3Cys, all potent TLR2 ligands, at doses that induced TNFα production, were unable to induce LDs biogenesis, suggesting that TLR2 activation although essential for mycobacterial-induced LDs is not sufficient to trigger pathways of LDs biogenesis and that other co-factors may be involved (Almeida et al., 2009). Additionally, the induction of LDs by *M. leprae* was significantly inhibited in both TLR2 and TLR6-deficient macrophages (Mattos et al., 2011a). However, TLR2 and TLR6 deletions affected LDs biogenesis in bacteria-bearing cells only partially, suggesting the involvement of alternative co-receptors. TLR6 deletion, but not TLR2, completely abolished the induction of LDs by *M. leprae*, as well as inhibited the bacterial uptake in Schwann cells (Mattos et al., 2011a).

The activation of TLR signaling induces the increased expression of several enzymes involved in the synthesis of triglycerides and/or cholesterol ester (Nicolaou et al., 2012). When *de novo* lipid synthesis is blocked, the biogenesis of LDs downstream of TLR activation is severely impaired during *M. leprae* infection (Mattos et al., 2011a).

The increase in the lipid content in the cells is accompanied by an upmodulation in the expression of the LD structural proteins, mainly PLIN2 (Gao and Serrero, 1999) and PLIN3 (Brasaemle, 2007). Among these regulators, PPARs have been the most explored during the mycobacterial infection. PPARγ has direct impact on LDs biogenesis by modulating PLIN2 expression, in non-adipocyte cells (Imamura et al., 2002; Larigauderie et al., 2004). The PLIN2 gene has a response element for PPARs and its expression is positively regulated by their agonists (Targett-Adams et al., 2005).

Almeida et al., have demonstrated that BCG infection is able to upregulate the expression and/or activation of PPARγ inducing lipid-laden macrophages, a phenotype that is inhibited when infected cells are pretreated with a selective PPARγ antagonist (GW9662). In addition, TLR2 stimulation by BCG triggers expression and activation of PPARγ and NF-κB. However, the downmodulation of PPARγ by GW9662 inhibits LDs biogenesis, while JSH-23, a specific NF-κB inhibitor, does not have effect on LDs biogenesis (**Figure 2**) (Almeida et al., 2009).

Moreover, it has been demonstrated that LDs biogenesis, lipid mediator production and PPARγ expression during BCG infection require the presence of CD36 together with TLR2, suggesting that cellular activation is a result of synergic interactions among these receptors (Almeida et al., 2014). Noteworthy, beyond TLR2 and CD36, blockade of CD14 and CD11b/CD18 also inhibited BCG-induced LDs biogenesis and the synthesis of lipid mediators, suggesting that in addition to CD36 other TLR2 co-receptors may regulate mycobacterial infection-triggered alterations in host lipid metabolism (Almeida et al., 2014).

CD36 can integrate cell signaling and metabolic pathways through its dual functions thereby influencing immune cells differentiation and activation. It is also involved in a wide range of functions, including uptake of oxidized low-density lipoprotein (OxLDL) and in bacteria binding DAMPS/PAMPS (Silverstein and Febbraio, 2010; Wang et al., 2020). In monocytes, CD36 is upregulated by PPARγ, as well as by cytokines including macrophage-CSF, IL-4, and IL-10 (Tontonoz et al., 1998; Chen et al., 2022). In mycobacterial infection, CD36 may participate in the entry of *Mtb* in adipocytes (Neyrolles et al., 2006). During BCG infection, Almeida et al. (2014) observed an upmodulation of CD36 expression in macrophages followed by an increase of LDs, indicating that CD36 may act in favor of the infection. Moreover, BCG infection *in vitro* is able to induce TNFα in macrophages but treatment with anti-CD36 antibody does not interfere in the synthesis of TNFα (Almeida et al., 2014). It is known that CD36 heterodimerizes with TLRs in lipid rafts. In fact, CD36 can co-immunoprecipitate with TLR2 in macrophages after 24 h of BCG infection differently from non-infected cells. Moreover, CD36 neutralization decreases PPARγ expression during BCG infection *in vitro* (Almeida et al., 2014).

In addition, KO mice with CD14 receptor deficiency are resistant to *Mtb* chronic infection whereas the wild-type mice are not. These data suggest that during chronic infection CD14 KO mice are protected from lethality caused by lung TB due to a reduction in the inflammatory response (Wieland et al., 2008). Furthermore, it has been described that THP-1 cells stimulated with LAM induced TNFα and IL-1β synthesis. However, this effect was inhibited by treatment with anti-CD14 monoclonal antibodies (Zhang et al., 1993). In fact, CD14 is able to interact with LAM and leads to IL-8 secretion in macrophages (Pujin et al., 1994). This data is corroborated by Almeida et al., 2014, who showed that both the anti-CD14 antibody treatment or CD14 KO peritoneal macrophages decreased LDs biogenesis after BCG infection.

Hence, these studies provide novel evidence that the activation of CD36, TLR2, CD11b/CD18 and CD14, compartmentalized within lipid rafts, induce the PPARγ activation which leads to LDs biogenesis through NF-κB-independent pathways. In addition, during BCG-infection, the rafts integrity is important for LDs biogenesis and lipid mediator synthesis, suggesting that TLR2 and co-receptors are present and interact in membrane domains to modulate host cell lipid metabolism and inflammatory response (Almeida et al., 2014).

## BCG-induced LDs sites for eicosanoid production

Eicosanoids are biologically active lipid mediators derived from the metabolization of fatty acids, mainly arachidonic acid (AA). Eicosanoids function as paracrine mediators of the inflammatory response, as well as intracellular mediators, playing a central role in the pathogenesis of various inflammatory conditions (Serhan, 1994; Bozza et al., 2011) Prostaglandins, leukotrienes (LT), thromboxanes, and lipoxins are examples of eicosanoids that show increased synthesis in inflammatory and infectious diseases such as asthma, rheumatoid arthritis, leprosy, TB and others (Bozza and Viola, 2010; Mattos et al., 2011b). Several immunosuppressive effects have been described for PGE_2_ at high concentrations, such as inhibition of Th1 profile cytokines as well as TNFα and nitric oxide synthesis, which are essential for the killing of mycobacteria (Aronoff et al., 2004; Serezani et al., 2007).

LDs have been implicated as important storage sites for AA, the substrate for eicosanoid synthesis (Weller and Dvorak, 1985; Dvorak et al., 1993; Weller et al., 1999). In addition, LDs are sites for eicosanoid-forming enzymes, such as 5-lipoxygenase (5-LO), LTC4-synthase and cyclooxygenases 1 and 2 (COX-1 and COX-2) (**Figure 1**), PGE_2_ synthase and others (Bozza et al., 1997; Silva et al., 2009; D’Avila et al., 2012). The utilization of AA stored in LDs was investigated by Eicosacell. In this technique, the EDAC fixative was used to cross-link eicosanoid carboxyl groups to amines in adjacent proteins, and to immobilize PGE_2_ demonstrating synthesis of PGE_2_ in BCG-induced LDs (D’Avila et al., 2006). In this perspective, we believe that LDs could function as a compartment for the rapid mobilization of AA for the synthesis of lipid mediators during mycobacterial infection (D’Avila et al., 2021).

An inhibition of LDs biogenesis was also observed when non-steroidal anti-inflammatory drugs, such as aspirin and NS-398, were used, accompanied by a decrease in PGE_2_, IL-10 and an increase in TNFα synthesis (D’Avila et al., 2006). Moreover, Almeida et al., have observed a decrease in bacterial viability when using the GW9662. This phenomenon may be related to the inhibition of LDs and PGE_2_ production by the GW9662 during BCG infection (Almeida et al., 2009) (**Figure 2**).

Moreover, in the *M. leprae* infection model, a decrease in LDs formation was observed during the pretreatment of the cells with C75 (inhibitor of the enzyme fatty acid synthase). This event was accompanied by the inhibition of PGE_2_ and IL-10 synthesis, together with an increase in IL-12 in Schwann cells (Mattos et al., 2011a). In these studies, an increase in the *M. leprae* mortality was observed after pretreatment of Schwann cells with NS-398 and C75. Furthermore, suppression of PGE_2_ in *Mtb* infected mice decreases bacterial replication and upmodulates IFNγ, TNFα and INOS (Moreno et al., 2002; Peyron et al., 2008). Thus, this increased ability of macrophages to produce PGE_2_ derived from LDs during mycobacterial infection might be related to the inhibition of a Th1 response, and consequently modulates negatively the macrophage activation, favoring pathogen survival and replication within the phagocyte.

## LDs-phagosome association during mycobacterial infection

Some species of mycobacteria are able to modulate the host cell’s endocytic pathway as an escape mechanism from intracellular degradation (Armstrong and Hart, 1971; Baltierra-Uribe et al., 2014). An important common feature of *Mtb* and BCG is their ability to retain the Rab5 protein in the phagosome, preventing Rab7 from being exchanged and, consequently, phagosomal maturation (Via et al., 1997; Fratti et al., 2001). It has also been described that Rab7 protein plays a key role in coordinating the anchoring and fusion of a number of vesicles and organelles (Jordens et al., 2001; Harrison et al., 2003). The immunofluorescence analysis showed the Rab5 protein to be close to LDs, but not co-localizes with these organelles. Nevertheless, when Rab7 protein was investigated, it colocalizes with the LDs (Roque et al., 2020). Similar results of Rab7 colocalization on LDs were observed in the hepatocytes subjected to nutrient deprivation. Nonetheless, in this uninfected context, the authors suggest that Rab7 acts as an important protein for lipolysis of LDs in cells under nutritional stress (Schroeder et al., 2015).

By ultrastructural analysis, it was observed intense accumulation of Rab7 in the contact site of LD and phagosome containing BCG, suggesting that LDs could be accumulating Rab7, contributing to inhibition of phagosomal maturation or acting as a regulator for vesicle fusion (Roque et al., 2020). Additionally, to complete the subsequent steps of the endocytic pathway to the phagosomal maturation, Rab7 must bind to its effector protein, RILP (Jordens et al., 2001; Harrison et al., 2003).

RILP is a protein responsible for recruiting the microtubule-associated motor complex, dynamin-dynactin, and has an important function in the fusion of phagosomes with lysosomes. Furthermore, studies have characterized RILP as a marker of Rab7 protein activation due to its ability to bind to the GTP only when it is in its active form (Cantalupo et al., 2001; Harrison et al., 2003).

Immunofluorescence results have revealed that Rab7 present on LDs is in its active form due to the presence of the RILP protein found in these organelles in experimental BCG infection *in vivo* (**Figures 1**, **2**) (Roque et al., 2020). Similar analyses were performed by Schroeder et al. (2015) where the presence of active Rab7 was observed analysing RILP in purified LDs from HuH7 cells (Schroeder et al., 2015). These data suggest that LDs induced by BCG infection may be sequestering active Rab7 in order to prevent phagosomal maturation, and/or being responsible for centripetal movement between the two organelles through binding with its effector protein, RILP (Roque et al., 2020). Harrison et al. (2003), described the role of RILP in the extension and fusion of tubules formed between organelles of the endocytic pathway (late phagosomes and lysosomes) of RAW 264.7 cells stimulated with opsonized latex particles (Harrison et al., 2003). Thus, the presence of Rab7 and RILP on LDs are indicative of the association between LD and phagosome during the endocytic pathway in infected cells. In these situations, LDs are retaining proteins important for phagosomal maturation and can act as an escape mechanism, favouring mycobacterial replication.

CID1067700, the pan GTPase Rab7 inhibitor, (Agola et al., 2013; Hong et al., 2015) was used to investigate the role of Rab7 in the interaction of phagosomes-LDs. This treatment with CID1067700 was able to completely inhibit the co-localization of phagosomes containing the LAM (from *Mtb*)-coated latex particles with the LDs (Roque et al., 2020). Corroborating with these data, a study demonstrated that inhibition of Rab7 by siRNA from Hep3B cells was able to prevent the recruitment of multi vesicular bodies (MVB) near the LDs in the nutritional deprivation-induced autophagy model (Schroeder et al., 2015).

Association of phagosomes-LDs have also been described in infections with *M. leprae*. Mattos et al., 2011, described in a model of *M. leprae* infection in Schwann cells the recruitment of LDs to the mycobacterium-containing phagosome in a mechanism dependent on microtubules and actin filaments. The proximity between phagosomes containing *M. leprae* and LDs was inhibited after treatment of the cells with taxol (microtubule stabilizer), or cytochalasin D (inhibitor of actin polymerization) or LY294002 (inhibitor of PI3K) without inhibiting the LDs biogenesis. Moreover, the inhibition of the association of phagosome-LDs treated with taxol, or cytochalasin D, or LY294002 decreased bacterial viability, suggesting a role for this interaction in favour of *M. leprae* infection (Mattos et al., 2011a; Mattos et al., 2011b).

Similarly, Russell et al. (2009) also observed not only the interaction between these organelles, but also the presence of a phagosome containing *Mtb* inside LD at advanced stage of infection. Additionally, Mattos et al. (2011b) also observed *M. leprae* embedded in the lipid content present in the phagosome. These data show there can be an exchange of host-bacterial lipids due to the interaction with the LDs corroborating that host lipids may be metabolized and used by the mycobacterium acting as a carbon source or even acting as armor protecting the bacterium from the host immune system (Pandey and Sassetti, 2008; Mattos et al., 2011b; Roque et al., 2020).

Nonetheless, the presence of LAM from BCG co-localized with some LDs *in vitro* (Roque et al., 2020). LAM has been described as a component of the mycobacterial wall with a key role for the survival of the bacillus inside cells (Hayakawa et al., 2007). LAM-coated latex particles or LAM from BCG actively approach phagosomes-LD in a Rab7 dependent mechanism (Roque et al., 2020). This feature corroborates the Beatty et al., study who demonstrated the trafficking of BCG cell wall lipids from infected phagosomes to other organelles during infection in macrophages *in vitro* (Beatty et al., 2001).

In summary, these data suggest the role of LDs phagosome interaction in preventing its maturation by sequestering the Rab7 protein and as sources of nutrients for the bacteria, thus favouring pathogen survival.

## Final remarks and perspectives

Although there have been great advances in recent years to characterize the mechanisms of LD biogenesis and functions during mycobacterial infection, there is still much controversy over whether LDs promote the host resistance to infection, or the bacterial escape mechanism, favoring the host susceptibility to infection. Recently, studies have shown that *Mtb* can persist in the cell, which is related to the activation state and metabolic pathways of its host (Genoula et al., 2020). The results of this study indicated that LDs accumulation mediated by STAT6 in foam macrophages is beneficial to *Mtb* persistence and promotes the further development of TB (Genoula et al., 2020).

However, Agarwal et al. (2020), suggested the potential effect of fatty acid supplementation to modulate anti-TB responses. In this study, they suggested that the decreased phagocytic capacity of foam cells increased survival and inflammatory potential against *Mtb*, altering the outcome of infection in patients with reduction of transmission. Therefore, foam cell formation reduces the host cell avidity for phagocytosis of *Mtb* while protecting the cells from death (Agarwal et al., 2020). This protective effect is associated with enhanced inflammatory potential of foam cells and restricted intracellular growth of *Mtb*. Furthermore, recent studies indicate that LDs induced by infection could sequester cytotoxic compounds (such as antimicrobial molecules), preventing damage to other cellular organelles, or reprogramming cell metabolism (Bosch et al., 2020). Other authors suggest that LDs can accumulate lipophilic antibiotics and then transfer to compartments containing the pathogen (Dubey et al., 2020; Greenwood et al., 2020).

Knight et al. (2018), proposed that LDs biogenesis in macrophages might not be an *Mtb*-induced process but that it could be a glycolytic programming event dependent on IFNγ and HIF1-α signaling in murine macrophages. In this same study, IFNγ-induced LDs were an important platform for the production of the host protective eicosanoids, such as LTB_4_ and PGE_2_, improving the macrophage immune response. However, the presence of IFNγ is not sufficient to induce LDs biogenesis, requiring a TLR2 activation as a second signal. This fact does not exclude the *Mtb* as a possible inducer of LDs biogenesis. Interestingly, *Mtb* was able to acquire host lipids in the absence of LDs but not in the presence of IFNγ-induced LDs (Knight et al., 2018).

Nonetheless, several studies have shown that usurpation of fatty acids released from the host triglycerides stored in LDs is a vital source of energy for mycobacteria and that the storage of fatty acids in the form of triglycerides in bacterial LD could be linked to the dormancy and reactivation of *Mtb* (Peyron et al., 2008; Russell et al., 2009). Cholesterol is another essential lipid to mycobacterial survival. *Mtb* has the capacity to use cholesterol as an energy source, which is important during the latent-phase of the infection (Pandey and Sassetti, 2008). While it seems contradictory to current literature, the induction of LDs as a host response cannot be underestimated, suggesting that the LD is an early protective organelle of the host. In fact, future studies are required to characterize these complex interactions according to the different pathogens.

## Conclusion

The formation of LDs in host cells during mycobacterial infections is a frequent phenomenon, which happens in a mechanism highly regulated by cellular receptors, such as TLRs, scavenger receptors, and PPARγ. In this mini-review we discussed some of the typical functions of LDs, such as acting in the supply of nutrients for the pathogen, inducing the synthesis of PGE_2_ which negatively modulates the microbicidal function of macrophages, as well as the regulation of the endocytic pathway. Furthermore, we discuss the controversial role of LDs, which may act in the host defense mechanism, as an intracellular source of antibiotics and as microbicidal molecules which are involved in the pro-inflammatory response. Thus, LDs may work as important targets for the development of new strategies for the control of mycobacterial diseases such as TB.

## Author contributions

HD and PA drafted the manuscript. PR and RS edited figures. HD, NP, PR, RS, JC and PA wrote and approved the final version of the paper. PA and HD edited the manuscript. All authors contributed to the article and approved the submitted version.
